# Insights into Systemic Disease through Retinal Imaging-Based Oculomics

**DOI:** 10.1167/tvst.9.2.6

**Published:** 2020-02-12

**Authors:** Siegfried K. Wagner, Dun Jack Fu, Livia Faes, Xiaoxuan Liu, Josef Huemer, Hagar Khalid, Daniel Ferraz, Edward Korot, Christopher Kelly, Konstantinos Balaskas, Alastair K. Denniston, Pearse A. Keane

**Affiliations:** 1 NIHR Biomedical Research Center at Moorfields Eye Hospital NHS Foundation Trust and UCL Institute of Ophthalmology, London, UK; 2 Department of Ophthalmology, Cantonal Hospital Lucerne, Lucerne, Switzerland; 3 Department of Ophthalmology, University Hospitals Birmingham NHS Foundation Trust, Birmingham, UK; 4 Academic Unit of Ophthalmology, Institute of Inflammation & Ageing, University of Birmingham, Birmingham, UK; 5 Google Health, London, UK

**Keywords:** deep learning, artificial intelligence, optical coherence tomography

## Abstract

Among the most noteworthy developments in ophthalmology over the last decade has been the emergence of quantifiable high-resolution imaging modalities, which are typically non-invasive, rapid and widely available. Such imaging is of unquestionable utility in the assessment of ocular disease however evidence is also mounting for its role in identifying ocular biomarkers of systemic disease, which we term *oculomics*. In this review, we highlight our current understanding of how retinal morphology evolves in two leading causes of global morbidity and mortality, cardiovascular disease and dementia. Population-based analyses have demonstrated the predictive value of retinal microvascular indices, as measured through fundus photography, in screening for heart attack and stroke. Similarly, the association between the structure of the neurosensory retina and prevalent neurodegenerative disease, in particular Alzheimer’s disease, is now well-established. Given the growing size and complexity of emerging multimodal datasets, modern artificial intelligence techniques, such as deep learning, may provide the optimal opportunity to further characterize these associations, enhance our understanding of eye-body relationships and secure novel scalable approaches to the risk stratification of chronic complex disorders of ageing.

## Introduction

The convergence of modern multimodal imaging techniques and large-scale data sets has fostered an extraordinary opportunity to exhaustively characterize the macroscopic, microscopic, and molecular ophthalmic features associated with health and disease (i.e., the *oculome*). One of the potential avenues of this *oculomics* revolution is the leveraging of the retina to gain insights beyond the eye. As the only human tissue allowing direct noninvasive in vivo visualization of the microvascular circulation and central nervous system, the neurosensory retina affords a unique setting for the characterization of systemic disease. Microvascular changes precede clinical manifestation, and so their detection should have predictive value.^1^ Indeed, ophthalmoscopic changes in retinal microvasculature structure have been identified as independent predictors for hypertension, diabetes, coronary disease, renal disease, and stroke.^2^^–^^6^ Alterations in the thickness of the retinal nerve fiber layer and macular volume, most easily revealed through optical coherence tomography (OCT), may highlight those individuals most at risk of developing cognitive decline and neurodegenerative disease.^7^^–^^10^ Furthermore, certain disorders may exhibit distinct retinal manifestations signifying their presence—the sea fan neovascularization of sickle cell anemia, the macular crystals of cystinosis, or the astrocytic hamartomas of tuberous sclerosis.^11^

A major facilitator for this has been the advancement in retinal imaging modalities over the past two decades. Primitive methods of direct ophthalmoscopy have evolved to encompass a diverse armamentarium of high-resolution imaging techniques, which are predominantly easy to acquire, risk free, and often demanding only nominal expertise and time for acquisition. In particular, both modern retinal photography and OCT are now of unprecedented resolution and increasingly carried out on a routine basis in both hospital eye settings and within the community.^12^ At Moorfields Eye Hospital NHS Foundation Trust, the largest ophthalmic unit in North America and Europe, we have seen a 14-fold increase in the capture of OCT from 23,500 scans in 2008 to >330,000 in 2016.^13^ Similarly, the availability of such cross-sectional imaging has exploded in primary care settings—the largest optical franchise in the United Kingdom, Specsavers, announced in 2017 that each of its >700 branches would have an OCT device by the end of 2019.^14^ The primary objective of this transformation has been to enhance the diagnosis of sight-threatening retinal disease, but an important parallel opportunity is emerging—the ability to use ocular biomarkers to detect systemic disease, predict its future onset, and provide noninvasive surrogates of its severity and treatment response.

Meaningful quantitative relationships between retinal structure and systemic disease have now been established using population-based analyses in cardiovascular disease (CVD) and dementia. In the former, changes in retinal microvasculature, from vascular caliber to tortuosity indices, have been associated with CVD risk factors and may have predictive value for relevant events, such as myocardial infarction and stroke.^4^^,^^5^^,^^15^^–^^17^ Traditionally, this has relied upon onerous manual segmentation of digitized images. The development of semiautomated retinal imaging analysis software has alleviated this burden but still demands significant time and researcher input for large-scale data sets highlighting the requirement for fully automated means, which may be addressed by modern methods of artificial intelligence (AI). One form of AI, deep learning, may be the answer. In 2018, researchers at Google Brain not only constructed a model capable of predicting CVD risk factors with reasonable accuracy but, more surprisingly, was also able to predict age and sex with impressive confidence.^18^ Reassurance of the rationale behind the model's output came from interpretability techniques highlighting retinal vasculature, the optic nerve, and macular morphology in decision making.

Although our understanding of eye-body relationships has evolved from decades of traditional statistical modeling in large population-based studies incorporating ophthalmic assessments, the application of AI to this field is still in its early stages. In this article, we focus on where AI-based studies may build on these traditional analyses to reveal novel insights leveraging retinal biomarkers of systemic disease. Particular focus is paid to common chronic disorders of aging, such as cardiovascular and neurodegenerative disease.

## Cardiovascular Disease

The first reported association between systemic vascular disease and the eye likely comes from the British nephrologist, Richard Bright, who in 1836 described a series of patients with albuminuria and vision loss.^19^ The ophthalmic features of what would become known as Bright disease, an umbrella term for all forms of glomerulonephritis, would not be rationalized until after the invention of the ophthalmoscope, when Marcus Gunn noted features of severe hypertensive retinopathy in a cohort of patients with chronic renal impairment in 1892.^20^ The concept of retinal-based quantification of cardiovascular disease risk would subsequently come in 1939, when Keith et al.^21^ would describe their prominent grading system for hypertensive retinopathy, which “permitted an intelligent appraisal of the individual patient and has increased considerably the accuracy of prognosis.”

As CVD is the leading cause of global mortality, accounting for more than 30% of deaths worldwide, there has been significant motivation to develop effective tools to identify those most at risk.^22^ The 2019 guidelines from the American College of Cardiology and American Heart Association recommend use of the ASCVD Risk Estimator Plus, which calculates a 10-year CVD risk score based on certain risk factors (age, sex, ethnicity), bedside tests (e.g., blood pressure), and blood parameters (e.g., total cholesterol).^23^ However, even such risk stratification algorithms can have limited calibration and discriminative ability when externally validated.^24^^,^^25^ Moreover, generating these scores depends on significant health care professional input and laboratory testing.

The use of a single noninvasive “eye check” to assess CVD risk is an attractive alternative, in part because of the importance that most of the population places on matters related to their vision and eye health. When surveyed, the general public ranks sight as our most important sense.^26^ This translates into significant differences between the extent to which members of the public attend eye checks compared to screening for CVD. For example, the free “Over-40” check established by the UK National Health Service for CVD risk stratification by primary care physicians in 2009 was attended by only 12.8% of the population from 2009 to 2013.^27^ In contrast, more than half the population attended their community optometric practice for regular eye checks in 2016.^28^

A role for retinal photography can therefore be envisaged in three settings. First, it could be used as an additional investigation, enabling risk refinement. There is evidence that the addition of retinal photography can positively affect reclassification indices for current risk stratification scoring systems.^29^^,^^30^ Its use as a mandated additional investigation would have significant resource implications, but an alternative would be the inclusion of data from imaging in those cases where it is available, in which case the requirement is primarily one of data integration. Second, there may be a role for retinal photography as an alternative to current CVD risk approaches in resource-poor settings. The emergence of widespread retinal photography in the developing world through smartphone technology and improved access for diabetic retinopathy screening may enable democratization of CVD risk stratification to this neglected population. Third, it may be used as an ad hoc screening test. Deployment in community optometry settings could allow identification of people at risk of CVD who would not otherwise have attended their primary care physician and are therefore prompted to have further investigation.

Direct noninvasive visualization of the microvascular circulation is a unique attribute of the neurosensory retina. The shared anatomical and physiologic characteristics between retinal vessels and those of the kidney and heart support the potential utility of retinal assessment as a conduit to systemic vascular disease risk stratification. The assessment of the retinal circulation has evolved from initially being through direct ophthalmoscopy, which is fraught with substantial intra- and interobserver variability, to the introduction of digital or digitized retinal photography with greater repeatability and precision.^31^^,^^32^ A large number of population-based studies have now demonstrated significant relationships between retinal vascular features and both cardiovascular disease outcomes, in particular myocardial infarction and stroke, and risk factors (age, blood pressure, smoking, presence of diabetes mellitus).^15^^,^^16^^,^^30^^,^^33^

The most convincing association has been made between risk of incident stroke and retinal vascular morphology. The Atherosclerosis Risk in Communities study was the first population-based study to evaluate the relationship between retinal vasculature and cardiovascular disease on a large scale and incorporate retinal photography.^15^ Not only were images graded for qualitative features suggestive of hypertensive retinopathy, such as cotton wool spots and arteriovenous nicking, but, following digitization of the images, semiquantitative measurement of the retinal vessel caliber also was completed. Controlling for known risk factors, including age, sex, diabetes, and blood features, most features of retinopathy were indeed associated with a higher relative risk of incident stroke, but it was also noted that such risk increased in those with smaller arteriole-to-venule ratios in a proportional scale. This was reinforced by similar results among diabetic patients in the Wisconsin Epidemiologic Study of Diabetic Retinopathy.^34^ However, interestingly, a meta-analysis incorporating six studies evaluating retinal vascular caliber and incident stroke over 5 to 12 years concluded that wider retinal venular caliber and not retinal arteriolar caliber predicted stroke with a pooled hazard ratio of 1.15 (confidence interval, 1.05–1.25 per 20-micron increase in caliber).^5^ To a certain extent, this finding persists when considering coronary heart disease (CHD) but with an important distinction. Retinal vascular morphology appears to be helpful only for risk stratification in women. A similar meta-analysis by McGeechan and colleagues^4^ evaluating the same six population-based cohorts revealed wider retinal venules, and narrower arterioles predicted 5- to 14-year risk of CHD events (namely, myocardial infarction, coronary bypass grafting, and coronary angioplasty) in women but not in men. The authors hypothesize that microvascular dysfunction plays a more prominent role in CHD risk in women.

Rather than focusing on CVD events, a number of studies have established links between retinal vascular characteristics and risk factors. In recent work using over 5000 participants from the European Prospective Investigation into Cancer-Norfolk Eye Study, Owen et al.^35^ examined retinal vessel caliber and tortuosity using the fully automated software QUARTZ. Increasing arteriolar tortuosity was associated with rising age and systolic blood pressure, whereas venular tortuosity was more related to body mass index and prevalent type 2 diabetes mellitus. In terms of vascular caliber, retinal venular caliber was higher in older patients, smokers, and those with raised triglyceride levels. Arterioles were narrower in older patients and those with higher systolic blood pressure and total cholesterol.

The studies mentioned thus far have centered on traditional statistical modeling techniques, such as regression and survival analysis, to draw insights on how retinal structure changes in CVD. However, these hypotheses-based methods rely on clinician direction within a narrow prespecified group of parameters. In contrast, Poplin et al.^18^ argue that the plethora of information within retinal photographs lends itself ideally to deep learning. The team from Google Research trained a convolutional neural network on fundus photos of >280,00 patients from the UK Biobank and EyePACS to predict CVD risk factors. Not only did the model indeed predict smoking status and major cardiac events with reasonable accuracy (area under the receiver operating characteristic curve [AUC] of 0.71 and 0.70 respectively), it surprised many readers in its ability to identify sex and age with high confidence (AUC of 0.97 and mean absolute error of 3.26 years, respectively). Importantly, the model performed well when externally validated on a separate data set of Asian patients by an independent research group.^36^ A further novel feature of their work reflects on the concern of the limited interpretability inherent in deep learning systems. They employed the deterministic technique, soft attention, to illustrate which pixels of the image were most influential in the model's decision. Although ophthalmic researchers may not be surprised to see that systolic blood pressure prediction was predominantly predicted using the retinal vessels, it may fascinate others to learn that the foveal appearance was instrumental in predicting biological sex. These saliency maps therefore have the potential to not only reassure us of the biological plausibility of model decision making but also stimulate research into potential novel biomarkers, akin to phenotype-first genome-wide association studies.

## Neurodegenerative Disease

There are over 9.9 million new cases of dementia worldwide each year. The World Alzheimer Report 2015 estimates that the number of affected people will double every 20 years, equating to >130 million cases in 2050.^37^ In the United Kingdom, dementia overtook CVD as the leading cause of death in 2016.^38^ Yet, despite these alarming figures, it has been estimated that 50% to 80% of cases of the most common form of dementia, Alzheimer disease (AD), remain unrecognized in high-income countries, due to the challenges in detection and diagnosis.^37^ Typically, individuals suspected of having dementia are assessed by their primary care physician using the usual combination of medical history, physical examination, and, in some cases, investigations in conjunction with a questionnaire regarding cognitive function. However, not only do these have variable diagnostic accuracy, but they also depend on an index of suspicion as well as attendance by the individual. In addition, the gold-standard diagnosis for some forms of dementia, such as AD, relies on brain biopsy, which is not appropriate for routine use. Evidence is growing for less invasive investigations such as cerebrospinal fluid analysis of amyloid protein and magnetic resonance imaging (MRI), but again, there are resource implications to using these tests at scale.^39^^–^^41^ One imaging protocol increasingly used for AD diagnosis, MRI–positron emission tomography amyloid, requires a prescan injection of a radioactive tracer and can take over an hour to complete. This is in stark contrast to the seconds needed for acquiring retinal imaging.

In 1986, following on from observations that people with AD had visual deficits that could not be explained purely by cortical disease, David Hinton and colleagues histologically examined the optic nerves of 10 patients with AD.^42^ Their report was the first to conclusively demonstrate the reduction in retinal nerve fiber layer and retinal ganglion cell number typical of AD. This is perhaps unsurprising given the shared embryologic origin of the cerebral cortex and eye. Emerging initially as diverticula in the primitive diencephalon in weeks 3 to 4 of development, the primitive optic vesicle undergoes a series of invaginations in tandem with overlying mesenchyme and surface ectoderm to result in a partition between the layers of the retina and walls of the forebrain bounded by the optic nerve. Accordingly, the cerebral cortex and eye share many characteristics, including immune privilege mediated through a combination of physical barriers, such as the blood-retinal barrier with its resemblance to the blood-brain barrier, and inhibitory microenvironments. Optic nerve morphology also mirrors that of its central nervous system counterparts ensheathed in myelin and three meningeal layers.

Before considering the relevant literature in this field, it is important to appreciate the semantic distinctions within neurodegenerative disease research, which may not be immediately obvious to visual scientists and ophthalmologists. Rather than describing a disease, the term *dementia* is an umbrella term referring to a constellation of symptoms secondary to impairment of higher-order cerebral functions such as memory, language, and problem solving. Accounting for >60% of cases of dementia in people older than 65 years, AD is the most common cause of dementia and is classically characterized by the deposition of amyloid beta oligomers and neurofibrillary tangles.^43^ In contrast, vascular dementia, the second leading cause, is intimately linked to cerebrovascular disease and can be subcategorized depending on the imaging characteristics of infarcts and white matter changes. The remaining ∼20% are attributable to rarer forms such as Lewy body dementia, frontotemporal degeneration, and dementia associated with Parkinson disease. A large proportion of cases may also be mixed, most commonly AD and vascular dementia, suggesting not only that both retinal vascular morphology and nerve fiber layer may be useful but that significant overlap exists in biomarker measurement.

Much of the current work bridging AD and retinal morphology revolves around OCT, but meaningful associations have been reported in other imaging modalities. As one of the first available modalities, retinal photography in those with cognitive decline showed changes in vessel caliber and branching indices, but this may be accounted for by the shared risk factors between CVD and certain forms of dementia. The Rotterdam Study demonstrates evidence of retinopathy in participants with prevalent dementia, but its presence did not appear to confer an increased risk of incident dementia.^44^ The peripheral retina may also hold clues—patients with AD appear to have both a higher baseline and an increase in peripheral hard drusen over two years of follow-up.^45^ Anatomically, however, changes in the optic nerve and retinal nerve fiber layer would seem most plausible representatives of neurodegenerative disease. Early on, it was noted that red free retinal nerve fiber layer (RNFL) photographs reveal a higher proportion of defects in patients with AD, but this has not always been consistent, perhaps owing to the limited number of cases in many studies.^46^ Trick et al.^47^ and Berisha et al.^48^ directly sought to address previous psychophysical work revealing inferior visual field defects in patients with AD, finding that the superior RNFL was preferentially impaired in their cohort.

The most consistent retinal feature of AD is OCT-measured changes in the RNFL. The RNFL comprises the axons of retinal ganglion cells that project directly to the lateral geniculate nucleus, and thinning has been shown to correlate with AD and cognitive decline in two separate meta-analyses.^7^^,^^10^ Individuals with AD show reductions in the RNFL, ganglion cell–inner plexiform layer, and overall macular volume.^49^ The similarity of RNFL thickness between people with established AD and mild cognitive impairment suggests that ganglion axonal loss occurs early in the disease and may therefore have some predictive value.^50^^,^^51^ In 2018, two large population-based analyses substantially bolstered our understanding in this field. A study by Ko et al.^9^ analyzing the OCTs of >30,000 participants of the UK Biobank study found that thinner RNFL was not only associated with lower cognitive testing scores (as measured by the Mini Mental State Examination), but also those in the thinner quintiles were more likely to perform poorly on the test three years later. In the same month, researchers from the Rotterdam Study published their findings that thinner RNFL was associated with an increased risk of developing dementia, highlighting the potential role of a biomarker of early dementia.^8^

To our knowledge, there is no published work relating to the use of deep learning in the prediction of dementia from retinal imaging, but this is likely to change soon given current research efforts. In a 2019 systematic review, Jo et al.^52^ appraised 16 studies employing deep learning in neuroimaging (magnetic resonance imaging) for the diagnosis of AD. It therefore seems likely that similar applications will pervade the retinal sphere.

## Prospects

We have sought to highlight the enormous potential of leveraging retinal biomarkers for the characterization of systemic disease, particularly those of rising prevalence within the aging population. Given the size of emerging data sets and complexity of imaging techniques, these benefits may be most effectively secured through modern AI techniques, such as deep learning.^53^ The role of deep learning in this field is likely to extend beyond simple risk prediction from an individual's retinal images. In an attempt to combat the “black box” issue of limited interpretability in deep learning, groups are leveraging methods such as saliency maps to highlight image regions/pixels that most contribute to the model's decision. Given the vast quantities of data incorporated within modern ophthalmic imaging, such as volumetric OCT scanning, the identification of novel biomarkers through these methods is attractive. Indeed, as per the work of Poplin et al.^18^ on the prediction of cardiovascular risk factors from retinal photography, foveal morphology appeared to be crucial in the model's decision of determining sex. This ability to generate new hypotheses is exciting and should then be validated on a separate data set. However, this deviation from traditional hypotheses-led research must be approached with caution, accounting for the well-known issues associated with data dredging, which frequented early genomics research.^54^

A further application for deep learning in this field comes from segmentation. Many OCT devices now come with preinstalled automated segmentation software, but these are generally designed for the identification of retinal layers, rather than disease features, and can be complicated by error. Rather than providing a global classification for an input image, deep learning can be employed to label each specific pixel. This can then be fed into a further neural network to provide an overall classification. De Fauw et al.^55^ employed this technique in OCT classification for macular diseases by designing an intermediate segmentation map, which was then analyzed by a further model to provide a classification and triage urgency. This cascade not only standardizes anatomical output independent of the acquiring device but also permits a degree of interpretability by the clinician, who can more easily discern, for example, the presence of subretinal fluid in a diagnosis of neovascular age-related macular degeneration. However, the segmentation map has significant value independent of a feed-forward process. Segmentation facilitates our ability to record change more accurately in retinal morphology and will likely be instrumental in dynamic risk prediction models, especially given that retinal imaging is frequently acquired on a regular ongoing basis. Incorporation of updated cardiovascular risk factors so as to illustrate a trajectory of change has shown promise over more conventional risk prediction methods, and this principle will likely extend to retinal biomarkers.^56^

Among the most crucial steps in constructing deep learning–based models is the acquisition and curation of a training data set of sufficient magnitude. These are typically derived from either large prospective observational cohort studies or retrospective real-world data. In the former, the data may be available to researchers either through open access (e.g., MESSIDOR: http://www.adcis.net/en/third-party/messidor/) or upon application (e.g., UK Biobank: https://www.ukbiobank.ac.uk/). In a recent systematic review evaluating 82 studies comparing the performance of deep learning models with health care professionals in disease classification from medical imaging, 25 studies leveraged data from open-access repositories.^57^ In the field of oculomics, retrospective real-world data can be a more challenging option as the desired labels (such as myocardial infarction within five years) will not align with the original purpose for capture, typically being eye disease. Moreover, ophthalmic care is often provided in standalone ophthalmic settings. In this situation, researchers may consider record linkage as a possible solution. We provide details of a case example of this in Box 1.

In this article, we have focused on two specific exemplars, cardiovascular and neurodegenerative disease, but there is emerging potential for generating novel hypotheses from linking large-scale ophthalmic data sets with other specialties. Such is the objective of initiatives such as Health Data Research UK (https://www.hdruk.ac.uk), which seeks to unite health care data nationally to facilitate innovative discovery with a strong underpinning of public involvement and engagement. AI-based methods in conjunction with interdisciplinary expertise will be crucial in tackling the future health care challenges of common chronic disorders of the body. Undoubtedly, oculomics will be one of the keys to these efforts.

Box 1. **Box 1. Case Example: AlzEye—Linking Ophthalmic Imaging and Systemic Disease Labels at Scale to Provide New Insights into Dementia (and Cardiovascular Disease)**

When trying to achieve the necessary scale of data for machine learning approaches, the use of routinely collected data is an attractive alternative to the high-cost, researcher-led data sets compiled through epidemiologic studies or biobanks. One of the aims of such an approach is to create virtual biobanks much cheaper than otherwise possible (arguably a “biobank-on-a-shoestring”) and which may indeed better reflect the population of interest (vs. the somewhat skewed population that has been observed in some biobank programs).

An example of this kind of approach is AlzEye, the United Kingdom's first and largest linkage of complex three-dimensional imaging data (fundus photographs and retinal OCT) to systemic health diagnostic codes for the purposes of exploring retinal ultrastructural associations and predictors of dementia and its subtypes. AlzEye depends on the combination of both local and nationally held data sets within the United Kingdom's National Health Service (NHS). Specifically, AlzEye is a pseudonymized data set linking retinal photographs and OCT scans of all patients older than 40 years attending Moorfields Eye Hospital NHSFT with Hospital Episode Statistics (HES), a national database consisting of all admissions, emergency attendances, and outpatient appointments in England. The appropriate use and linkage of such data depend on satisfying many criteria, including ethical approval, data security, and governance. Engagement with the public has been pivotal to the approach. We surveyed 483 participants to canvass public opinion on the use of eye scans for research and the acceptability of large data sets to identify patterns of systemic disease. Two members of the public sit on the AlzEye working group, and information regarding the study is outlined on the funding charity's website.

**Figure 1.**
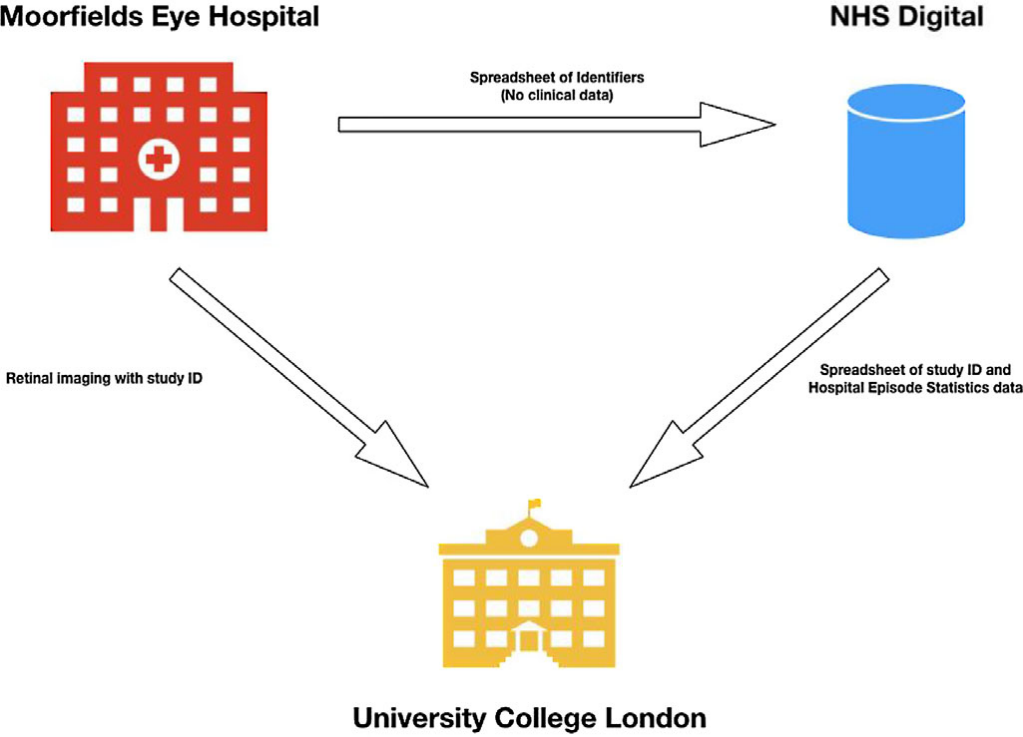
The flow of data is such that the Moorfields Eye Hospital never receives HES data and University College London does not receive any identifiers. University College London, as a trusted third party, links images from Moorfields Eye Hospital with HES data from NHS Digital based on a unique study ID.

This kind of study is complex, and the approval process that AlzEye underwent was appropriately robust with a number of different approvals required prior to the establishment of AlzEye. Although the exact process will vary from country to country, the processes are likely to share similar principles, and we therefore highlight them here. The first stage required us to secure a research sponsor, necessitating institutional approval consisting of research and development, information governance, and information technology at both the NHS data custodian (Moorfields Eye Hospital NHSFT) and the research institute (University College London). Important conditions involving third-party linkage by a “trusted third party,” robust data privacy measures, and sufficient computing infrastructure were outlined at this stage. In AlzEye, the linkage process is as follows: (1) images from Moorfields Eye Hospital are pseudonymized through the removal of all identifiers and replacement with a unique study ID. These are then transferred to University College London. (2) Simultaneously, a spreadsheet of the image identifiers (date of birth, unique NHS number, sex) is securely sent to NHS Digital, the national body overseeing the HES data warehouse. (3) NHS Digital strips the identifiers and returns the relevant HES data with pseudonymized study IDs to University College London, where it is linked with corresponding images. Thus, HES data never enter the source of imaging data (Moorfields Eye Hospital), and conversely, identifiers never enter University College London (Fig. 1).

Prior to commencement, all research studies in the United Kingdom require ethical approval through the Research Ethics Service, but some specific studies may warrant additional approvals. AlzEye was approved by the National Health Service Research Ethics Committee in 2018. Due to the large number of patients included (more than 250,000), the historical nature of the data, and the advanced age and difficulty in contacting patients, it would not be feasible to obtain consent from patients. Therefore, to use identifiable data for the linkage, a specific type of approval was sought involving an application to the Confidential Advisory Group, who advise the UK Health Research Authority on whether sufficient justification exists to access data without consent. In the United Kingdom, this is known as a “Section 251 approval,” deriving from the 2006 NHS Act, which provides provision for this kind of application. The Health Research Authority, collating the opinions of the respective committees, granted ultimate approval in late 2018.

Upon these approvals, applications to NHS Digital for the procurement of HES data can then be processed. In addition to the external approvals, NHS Digital has its own internal approval process detailing, in particular, the legal basis upon which data are being accessed. When a given application is approved, it is then presented on behalf of the applicant by NHS Digital to the Independent Group Advising on the Release of Data (IGARD), a committee of specialist and lay members who assess all applications to NHS Digital for the dissemination of confidential information. In January 2019, IGARD gave approval, remarking that the AlzEye application “could be used as an exemplar to help other researchers with their applications to the Data Access Request Service.”
